# A long-lived peptide-conjugated iridium(iii) complex as a luminescent probe and inhibitor of the cell migration mediator, formyl peptide receptor 2†
†Electronic supplementary information (ESI) available. See DOI: 10.1039/c8sc02733a


**DOI:** 10.1039/c8sc02733a

**Published:** 2018-10-01

**Authors:** Kasipandi Vellaisamy, Guodong Li, Wanhe Wang, Chung-Hang Leung, Dik-Lung Ma

**Affiliations:** a Department of Chemistry , Hong Kong Baptist University , Kowloon Tong , Hong Kong , China . Email: edmondma@hkbu.edu.hk; b State Key Laboratory of Quality Research in Chinese Medicine , Institute of Chinese Medical Sciences , University of Macau , Macao , China . Email: duncanleung@umac.mo

## Abstract

Formyl peptide receptors play important biological and therapeutic roles in wound repair and inflammatory diseases. In this work, we present a luminescent iridium(iii) complex (**6**) conjugated with the peptide agonist WKYMVm as a luminescent formyl peptide receptor 2 (FPR2) imaging probe in living cells. Complex **6** displayed ideal cell imaging characteristics, high photostability and low cytotoxicity. Competition assays with a known FPR2 antagonist, WRW4, and siRNA knockdown experiments both revealed that complex **6** selectively targeted FPR2 in living HUVEC cells. Moreover, complex **6** regulated FPR2 signalling in HUVEC cells as shown using a mechanical scratch assay. Finally, complex **6** reduced epithelial cell migration capacity and inhibited lipoxin A4 (LXA4)-triggered cell migration in HUVEC cells, demonstrating the ability of this complex to inhibit FPR2 in living cells. To our knowledge, this is the first long-lived probe for imaging FPR2 in living cells.

## Abstract 2

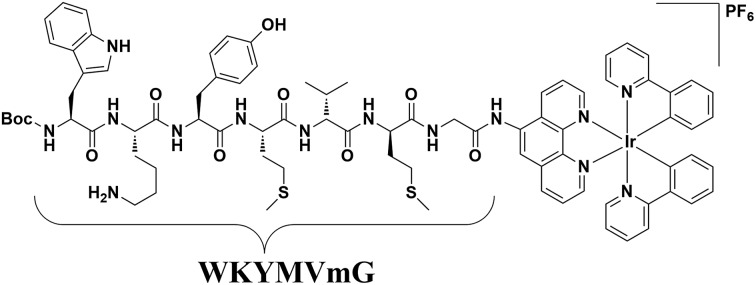
Formyl peptide receptors play important biological and therapeutic roles in wound repair and inflammatory diseases.

## Introduction

Leukocyte responses to chemotactic factors or chemoattractants during inflammation are dependent on specific G protein-coupled receptors.^1^ One class of these receptors is formyl-peptide receptors (FPRs), which include formyl peptide receptor 1 (FPR1), formyl peptide receptor 2 (ALX/FPR2 or FPR2), and formyl peptide receptor 3 (FPR3).^2^ FPRs facilitate the trafficking of phagocytes to the site of infection, and modulate the survival and the phagocytic activity of infiltrating cells. Moreover, FPRs play an important role in inflammatory angiogenesis.^3^ A cathelicidin peptide, LL-37, promoted angiogenesis in human umbilical vein endothelial cells (HUVECs) through an FPR2 signalling pathway.^4^ In liver epithelial cells, FPRs are capable of responding to mitochondrial antigens to enhance wound healing caused by linear scratch injury.^5^ In another study, annexin A1 (ANXA1) activated FPRs to repair intestinal wounds by a redox process.^6^ However, the understanding of FPR functions in wound healing is limited. Moreover, FPRs are emerging as therapeutic targets for a range of diseases, including cancer, inflammation, amyloidosis and neurodegenerative diseases.^7^ Therefore, the development of highly sensitive and selective probes for FPRs is required for further elucidating the biological and therapeutic roles of FPRs in wound repair and disease.

Noninvasive molecular-imaging techniques are powerful tools for studying biological processes in living systems.^8^,^9^ Peptide-based imaging probes have been increasingly studied due to their compatibility with living systems, high binding affinity, and adequate permeability.^10^,^11^ For FPRs, an FPR1 targetable cFLFLF peptide complexed with various radioactive metals (*e.g.* Ga(iii), In(iii), Gd(iii), ^99^Tc and ^64^Cu) has been widely employed for imaging of inflammation in various animal models.^12^–^16^ Peptide-based fluorescent probes have received considerable recent attention due to their high sensitivity, selectivity, and safe imaging performance.^17^ For example, peptide-based fluorescent probes have been reported for imaging matrix metalloproteinase (MMP)^18^,^19^ and cysteine protease.^20^–^22^ However, only a few peptide-based fluorescent probes have been reported for FPR1 16,23 and FPR2.^24^

Transition metal complexes are emerging as a promising class of luminophores for studying biological processes in living cells,^25^–^28^ due to their long emission lifetime, large Stokes shift, and high photostability. However, the poor cell permeability of transition metal complex-based probes still remains a challenge for their further bioimaging application. One effective strategy to overcome this bottleneck is the conjugation of a transition metal complex with a specifically targetable peptide.^29^,^30^ In recent years, considerable effort has been made to develop transition metal complex–peptide conjugates as imaging probes,^29^,^31^ such as cobaltocenium–peptide conjugates for studying cellular uptake,^32^ ruthenium-short peptide conjugates for nuclear staining,^33^ and ruthenium-cyclic RGD peptide conjugates for targeting integrin receptors.^34^ Considering the additional favorable characteristics possessed by iridium(iii) complexes including high quantum yields and tunable luminescence,^35^,^36^ the combination of an iridium(iii) complex and a peptide may provide additional synergism for bioimaging applications. However, peptide-functionalized iridium(iii) complexes as bioimaging probes have not been widely explored.^37^–^42^

**Fig. 1:**
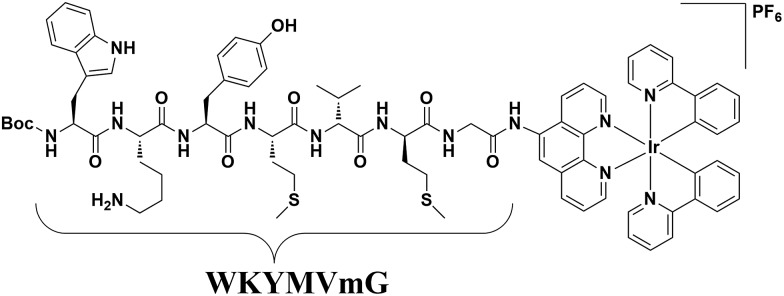
Chemical structure of the peptide iridium(iii) complex as an FPR2 probe.

**Scheme 1:**
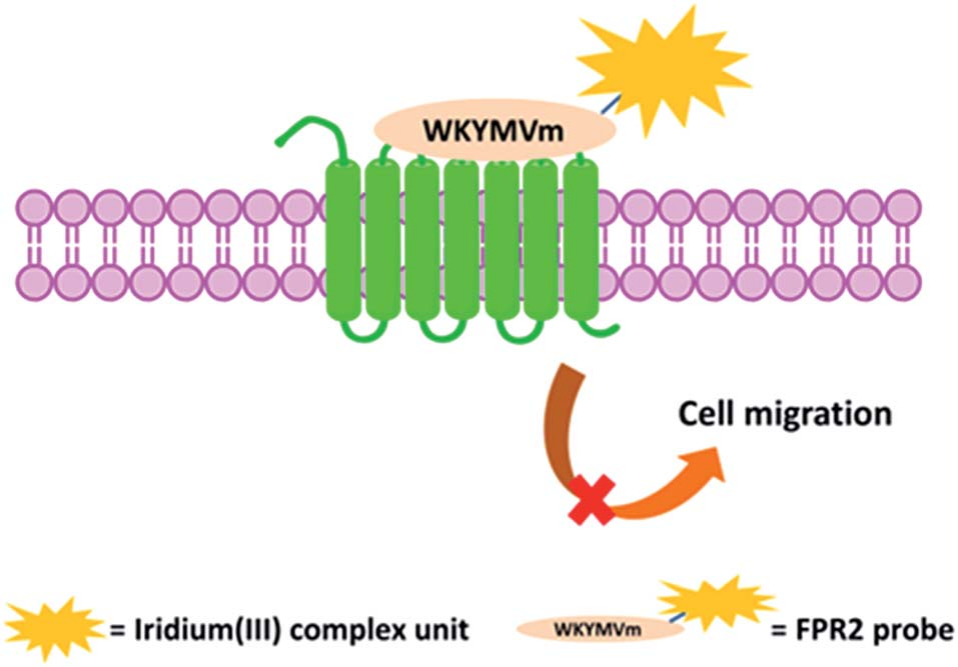
Schematic diagram of the peptide iridium(iii) complex as a luminescent probe and inhibitor of FPR2 in living cells.

The hexapeptide WKYMVm is a selective FPR2 agonist with high affinity, which was identified through screening of a synthetic peptide library.^43^,^44^ WKYMVm promoted recovery from tissue damage *via* an FPR2-dependent pathway in various disease models,^44^ such as cutaneous wound healing of diabetic mice,^45^ and a hindlimb ischemia model.^46^ In this study, we conjugated WKYMVm to a cyclometalated iridium(iii) complex to develop a new luminescent probe for FPR2 (Fig. 1). This peptide-conjugated cyclometalated iridium(iii) complex **6** visualized FPR2 in living cells. As complex **6** bears the peptide ligand (WKYMVm) agonist, we believe that the peptide-conjugated complex would not only be able to effectively recognize FPRs, but also regulate FPR2-mediated functions (Scheme 1).^47^ To our knowledge, this is the first report of an iridium(iii) complex for visualizing FPR2 in living cells.

## Results and discussion

### Synthesis of peptide-conjugated iridium(iii) complex **6**

**Scheme 2:**
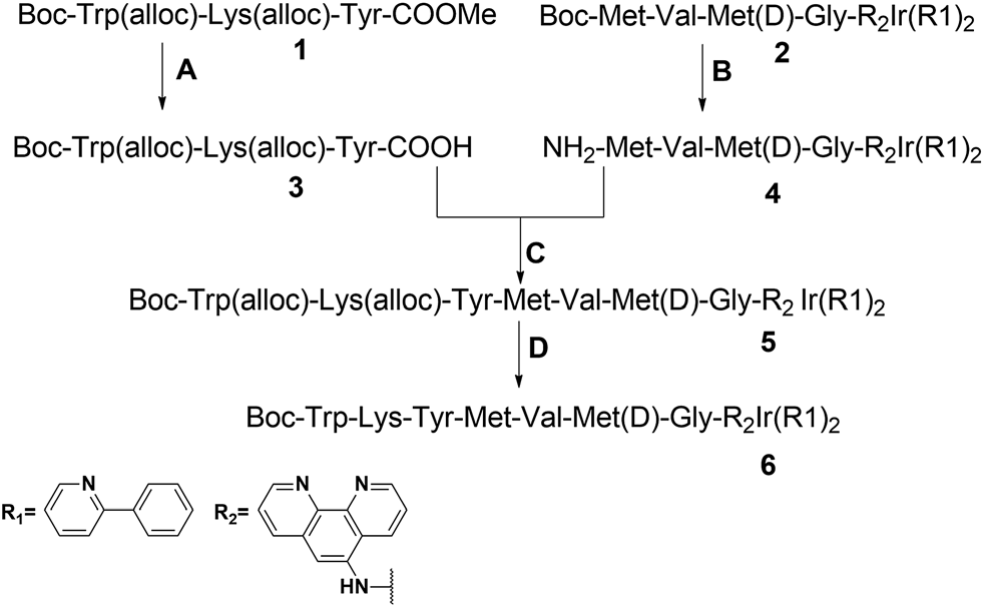
Synthetic route to the peptide iridium(iii) complex. Conditions: (A) LiOH, THF/H_2_O (1 : 1), 0 to r.t., yield = 95%; (B) TFA/DCM, quantitatively; (C) Et_3_N, EDCI, DCM, yield = 63%; (D) Pd/C, PhSiH_3_, MeOH, r.t., yield = 73% (alloc = allyloxycarbonyl).

The peptide-conjugated iridium(iii) complex **6** has the general structure of [Ir(C^N)_2_(N^N)](PF_6_) (where C^N = 2-phenylpyridine (ppy), and N^N = 1,10-phenanthrolin-5-amine-WKYMVmG). We envisaged that conjugating the WKYMVm agonist to an iridium(iii) scaffold could afford useful probes for FPR2. To synthesize the iridium(iii) complex **6**, the doubly allyl-protected tripeptide (Boc-Trp(Alloc)-Lys(Alloc)-Tyr-OMe) **1** was first synthesized using a standard solution phase peptide protection and deprotection strategy. Peptide **1** was hydrolyzed under basic conditions using lithium hydroxide (LiOH) in THF/water (1 : 1) to furnish the corresponding Boc-protected acid (Boc-Trp(Alloc)-Lys(Alloc)-Tyr-COOH) **3** (Scheme 2). Meanwhile, 2-amino-*N*-(1,10-phenanthrolin-5-yl)acetamide is coupled with the tripeptide Boc-Met-Val-d-Met-COOH. The resulting N^N ligand Boc-Met-Val-Met-Gly-1,10-phenanthroline was isolated in good yield (90%), and was complexed by reacting with a dimeric iridium precursor bearing 2-phenylpyridine (ppy) (C^N) ligands in dichloromethane (DCM)/MeOH (1 : 1) to generate the tetrapeptide-conjugated iridium(iii) complex **2**. Meanwhile, the tetrapeptide-conjugated iridium(iii) complex **2** was de-protected using trifluoroacetic acid (TFA) in dry DCM to generate the corresponding amine salt **4**. Subsequently, the Boc-protected acid **3** was coupled with amine **4** employing standard 1-ethyl-3-(3-dimethylaminopropyl)carbodiimide (EDCI) coupling conditions to yield the conjugated complex **5**. Finally, allyl carbomate deprotection of compound **5** with a catalytic amount of Pd/C/TES in methanol (MeOH) yielded complex **6** in good yield after silica gel column chromatography purification. The new complex **6** was fully characterized by ^1^H NMR spectroscopy and high-resolution mass spectrometry (HRMS) (Fig. S5–S7†).

### Luminescence profile of complex **6**

The photophysical properties of complex **6** were determined in acetonitrile (ACN) and are described in Table S1.† Notably, complex **6** showed a good quantum yield (0.245) and a long luminescence lifetime (4.62 μs). The UV-Vis absorption spectrum of complex **6** is presented in Fig. S8.† Complex **6** showed maximum excitation at 291 nm and maximum emission at 576 nm (Fig. S9†). The luminescence intensity of complex **6** was unchanged by the addition of 0.3 μg mL^–1^ FPR2 in PBS (pH 7.4) buffer (Fig. S10†). Moreover, complex **6** showed no response to common biological interferents in living cells, including amino acids, common metal ions (Zn^2+^, Mg^2+^, Ca^2+^, Fe^3+^ and Cu^2+^), and other small molecules or proteins (ATP, NADH and BSA) (Fig. S11†). Importantly, complex **6** showed excellent photostability, as luminescence intensity was not impaired even after 1800 s of continuous irradiation at 365 nm (Fig. S12†). Taken together, these results demonstrate the utility of complex **6** as a long-lived and permanent probe.

### Cytotoxicity and cell imaging of living cells

Considering the promising luminescence profile of the peptide-conjugated iridium(iii) complex **6**, its cytotoxicity was assessed in HUVEC cells using the 3-(4,5-dimethylthiazol-2-yl)-2,5-diphenyltetrazolium bromide (MTT) assay. Encouragingly, complex **6** showed an IC_50_ value of 63.09 μM even after 48 hours treatment (Fig. S13†), indicating that this complex is relatively nontoxic to HUVEC cells under the experimental conditions and rendering it suitable for cell imaging experiments. The cytotoxicity of compound **6** was further evaluated in three other cell lines, including a human hepatic cell line (LO2), a cervical cancer cell line (HeLa), and a breast cancer cell line (MDA-MB-231) using the MTT assay. Complex **6** showed low cytotoxicity to LO2, HeLa, and MDA-MB-231 cell lines with IC_50_ values of >100, >100, and 52.48 μM (Fig. S14†), respectively. Furthermore, to determine whether the conjugation of WKYMVm affects the toxicity of the iridium moiety, the cytotoxicity of the parent iridium(iii) scaffold **S1** (Fig. S15†) was examined. As shown in Fig. S16,† complex **S1** showed an IC_50_ value of 14.13 μM after 48 h treatment in HUVEC cells (*cf.* 63.09 μM for complex **6**). This result suggests that the incorporation of the peptide WKYMVm into an iridium(iii) scaffold can reduce the cytotoxicity of the metal moiety. Taken together, these results indicate that complex **6** is non-toxic enough to use for cell imaging experiments.

**Fig. 2:**
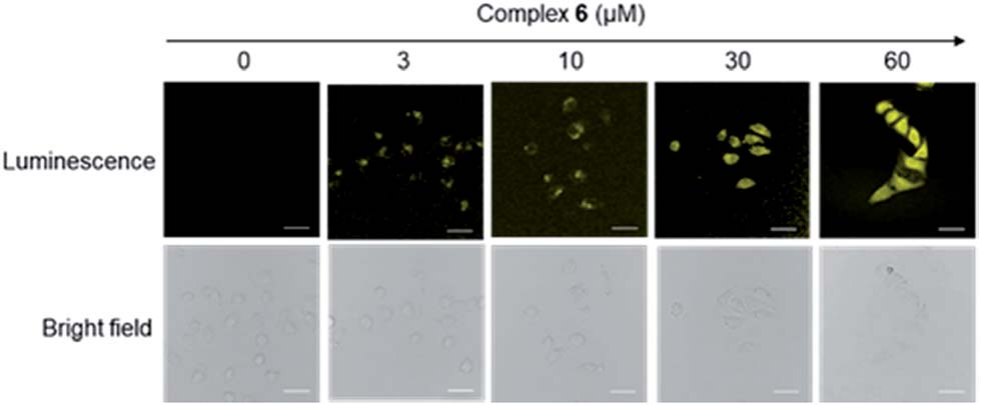
Luminescence and bright-field images of HUVEC cells incubated with 0 to 60 μM of complex **6** for 1 h. Scale bar = 50 μm.

We next studied the application of complex **6** for cell imaging. HUVEC cells, known to express FPR2,^48^ were incubated with complex **6** (0, 3, 10, 30 and 60 μM) for 1 h and then washed with phosphate buffer (PBS). Luminescence imaging was performed using confocal laser scanning microscopy with excitation at 488 nm. Encouragingly, the luminescence intensity of treated HUVEC cells increased with the dosage of complex **6** (Fig. 2).

**Fig. 3:**
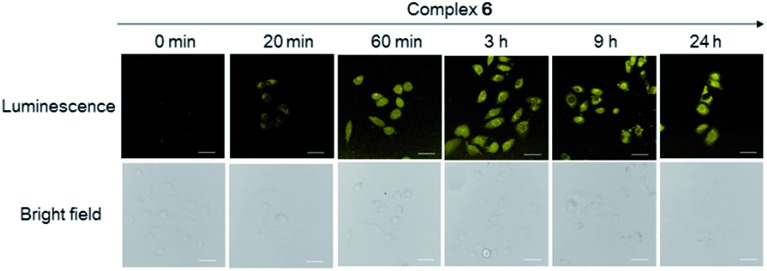
Luminescence and bright-field images of HUVEC cells incubated with 30 μM of complex **6** at different time intervals. Scale bar = 15 μm. HUVEC cell line stained by complex **6** at different incubation times (0 min, 20 min, 60 min, 3 h, 9 h and 24 h). Scale bar = 100 μm.

We presume that complex **6** could interact with FPR2 *via* its conjugated peptide agonist moiety, thereby resulting in an enhanced luminescence of the cells. A subsequent time-course experiment was performed to evaluate the kinetics of the luminescence response of complex **6** in living cells. The result showed that the yellow luminescence of complex **6** (30 μM) increased and reached a plateau within 1 h incubation in HUVEC cells. Thus, a reaction time of 1 h was chosen for subsequent experiments (Fig. 3).

### Validation of FPR2 binding in HUVEC cells

**Fig. 4:**
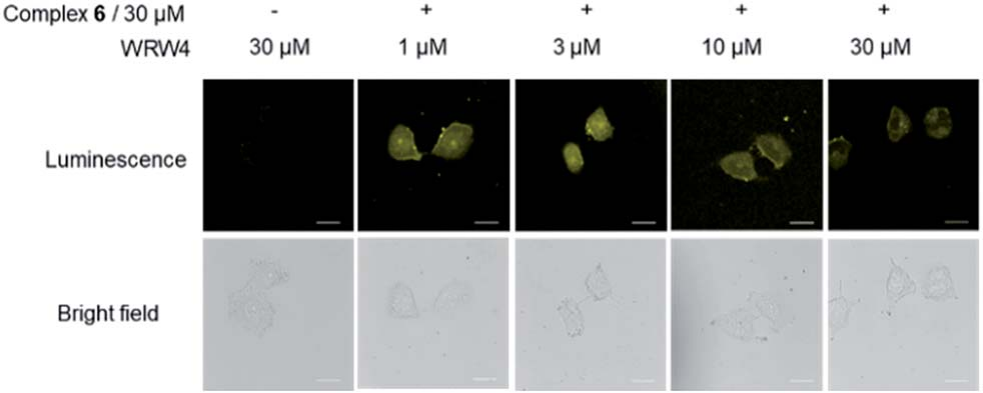
Luminescence and bright-field images of HUVEC cells. HUVEC cells were pretreated with or without WRW4 at various concentrations (1-30 μM) for 1 h, followed by staining with complex **6** (30 μM) for 1 h. Scale bar = 50 μm.

To provide further evidence that complex **6** interacted with FPR2 in living cells, we preincubated HUVEC cells with various concentrations of the FPR2-selective antagonist WRW4 (1, 3, 10 and 30 μM) for 1 h, before staining with complex **6** (30 μM) for 1 h. Notably, the luminescence intensity of complex **6** decreased in the presence of WRW4 (Fig. 4), which is presumably due to competitive binding to FPR2, thereby blocking the interaction between complex **6** and the receptor. To verify the role of the WKYMVm peptide in probe **6**, luminescence imaging experiments were also performed with the parent iridium(iii) scaffold **S1** and the tetrapeptide-conjugated complex **2** under the same conditions. The results showed that there was no significant change in the luminescence intensity of **S1** or **2** after pretreatment with WRW4 in HUVEC cells (Fig. S17†), suggesting that the WKYMVm peptide motif in complex **6** plays a key role in targeting FPR2 protein.

**Fig. 5:**
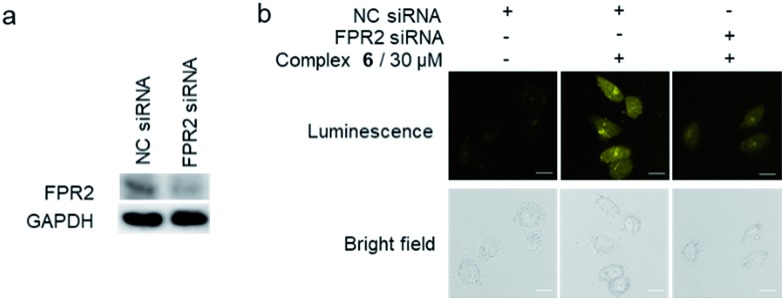
(a) FPR2 is down-regulated after FPR2 siRNA pretreatment. GAPDH was used as a loading control. (b) Normal HUVEC cells and FPR2 knockdown HUVEC cells were stained with or without complex **6** (30 μM) for 1 h. Scale bar = 50 μm.

To further verify the targeting of FPR2 by complex **6** in living HUVEC cells, siRNA knockdown experiments were employed. The levels of FPR2 reduced significantly after treatment of HUVEC cells with FPR2 siRNA (Fig. 5a). Next, complex **6** was introduced into FPR2 knockdown HUVEC cells. As shown in Fig. 5b, the luminescence of complex **6** reduced significantly, indicating that the emission intensity of the complex was correlated with the level of FPR2 in living cells. Taken together, these results suggest that complex **6** may act as an FPR2 imaging probe in living cells, allowing the visualization of FPR2 in a non-invasive manner and potentially enabling the visual study of its biological dynamics.^49^–^51^

### Complex **6** could inhibit the lipoxin A4 (LXA4)-triggered cell migration in HUVEC cells

Previous reports have shown that FPR2 activation could stimulate the invasion and migration of epithelial cells.^44^,^52^ Therefore, we employed the scratch assay to study FPR2's role in cell migration. The scratch assay was first performed in FPR2-silenced HUVEC cells to verify the role of FPR2 in cell migration (Fig. S18A†). Cell migration capacity was evaluated by quantifying the total distance that the HUVEC cells moved from the edge of the scratch (marked by imaginary horizontal lines) toward the center of the scratch.^53^ After treatment with negative control (NC) siRNA or FPR2 siRNA, the monolayers of HUVECs were scratched linearly with a 200 μL pipette tip, and images of wounded monolayer were taken at 12 h. The results showed that FPR2 silencing decreased the migration of HUVEC cells compared to cells treated with NC siRNA. This indicates that FPR2 is correlated with the cell migration capacity of HUVECs, which is consistent with previous reports.^54^,^55^

**Fig. 6:**
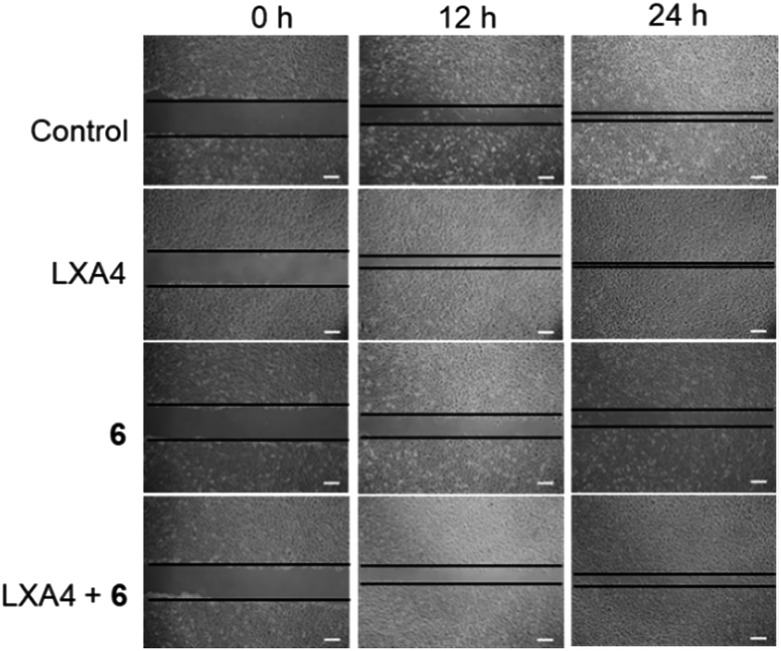
Complex **6** could inhibit the LXA4-triggered cell migration in HUVEC cells. Cell migration of epithelial cells grown as monolayers on plastics for 12 and 24 h after treatment with either ethanol control, LXA4 (1 nM), complex **6**, or complex **6** in the presence of LXA4. The horizontal lines indicate the wound edge. Scar bar = 200 μm.

To further evaluate the role of complex **6** as a regulator of FPR2 signaling in HUVEC cells, the scratch assay was repeated in the presence of complex **6**. As depicted in Fig. S18B,† complex **6** reduced the migration of HUVEC cells in a dose-dependent manner at 12 and 24 h, indicating that complex **6** may be a metal-based inhibitor of FPR2 in HUVEC cells. LXA4 is a bioactive eicosanoid with FPR2 agonist activity, triggering epithelial cell migration.^6^,^47^ The migration capacity of HUVEC cells treated with 1 nM of LXA4 was significantly enhanced compared to vehicle-induced HUVEC cells (Fig. 6). However, the LXA4-stimulated migration of HUVEC cells was significantly decreased in the presence of complex **6** (30 μM) after 12 h and 24 h. This result demonstrates that complex **6** is a potential regulator of FPR2-mediated functions in living HUVEC cells.

## Experimental

### Synthesis of compound **3**

Compound **1** was synthesised using standard solution phase peptide synthesis and it was characterised by NMR and HRMS. Compound **1**: ^1^H NMR (400 MHz, CDCl_3_) *δ* 8.15 (s, 1H), 7.58 (d, *J* = 7.7 Hz, 1H), 7.48 (s, 1H), 7.32 (dd, *J* = 16.5, 8.9 Hz, 2H), 7.23 (d, *J* = 7.3 Hz, 1H), 6.95 (d, *J* = 8.3 Hz, 2H), 6.76 (d, *J* = 8.0 Hz, 2H), 6.46 (s, 1H), 6.03 (m, 1H), 5.89 (d, *J* = 5.8 Hz, 1H), 5.59 (s, 1H), 5.46 (t, *J* = 3.9 Hz, 1H), 5.43 (d, *J* = 1.2 Hz, 1H), 5.36 (s, 1H), 5.33 (d, *J* = 0.9 Hz, 1H), 5.29 (d, *J* = 17.4 Hz, 1H), 3.70 (d, *J* = 10.2 Hz, 3H), 1.52 (s, 2H), 1.42 (s, 2H), 1.39 (s, 9H), 1.24 (d, *J* = 6.8 Hz, 3H). ^13^C NMR (101 MHz, CDCl_3_) *δ* 172.19, 171.74, 171.54, 156.00, 155.79, 155.58, 132.49, 131.38, 130.33, 127.04, 124.97, 123.78, 123.10, 119.59, 119.18, 118.11, 116.70, 115.69, 115.29, 80.52, 77.37, 77.25, 77.05, 76.73, 67.71, 66.02, 65.90, 54.74, 53.18, 52.52, 38.97, 36.80, 31.84, 28.30, 22.05, 15.31, 1.06. HRMS: calcd. for C_39_H_49_N_5_O_11_ [M + Na]^+^: 800.3477 found: 800.3446**.** Hydrolysis of the C-terminal methyl ester of compound **1** hydrolysed using LiOH in tetrahydrofuran (THF)/H_2_O (1 : 1) at 0 to r.t. gave the corresponding acid **3** in good yield (95%).

### Synthesis of compound **4**

The compound **2** peptide precursor was obtained using standard peptide coupling and the peptide precursor Boc-Met-Val-Met(d)-COOH was coupled with 2-amino-*N*-(1,10-phenanthrolin-5-yl)acetamide to give the corresponding peptide-conjugated ligand. The peptide attached ligand is reacted with the iridium-2-phenylpyridine dimer in MeOH/DCM (1 : 1) to get the corresponding peptide iridium(iii) complex **2**. Compound **2**: ^1^H NMR (400 MHz, DMSO) *δ* 10.45 (s, 1H), 8.97 (d, *J* = 8.8 Hz, 1H), 8.86 (d, *J* = 8.3 Hz, 1H), 8.60 (m, 1H), 8.24 (dd, *J* = 13.1, 6.6 Hz, 3H), 8.08 (m, 2H), 7.97 (m, 3H), 7.87 (dd, *J* = 11.0, 7.3 Hz, 2H), 7.73 (s, 1H), 7.45 (d, *J* = 5.6 Hz, 2H), 7.05 (t, *J* = 7.0 Hz, 3H), 6.95 (ddd, *J* = 12.0, 11.6, 6.0 Hz, 4H), 6.26 (t, *J* = 7.6 Hz, 2H), 4.41 (s, 1H), 4.15 (dd, *J* = 21.5, 6.8 Hz, 3H), 4.04 (s, 2H), 2.41 (s, 3H), 1.99 (m, 6H), 1.93 (s, 1H), 1.90 (s, 1H), 1.33 (m, 9H), 0.81 (s, 7H). ^13^C NMR (101 MHz, DMSO) *δ* 171.94, 171.72, 171.54, 170.99, 169.15, 166.83, 155.39, 150.74, 150.03, 149.68, 149.08, 146.54, 144.03, 143.97, 138.70, 138.28, 134.49, 133.67, 131.24, 131.19, 130.73, 130.22, 127.25, 127.08, 126.52, 125.06, 123.89, 122.39, 119.97, 78.25, 57.95, 57.56, 57.35, 57.22, 53.82, 53.57, 53.39, 52.91, 51.95, 51.91, 51.70, 42.92, 31.75, 31.61, 30.62, 29.69, 29.52, 28.12, 19.11, 17.75, 14.61, 14.56, 14.49. HRMS: calcd. for C_43_H_29_F_10_IrN_5_O_3_P [M – PF_6_]^+^: 1214.5031 found: 1214.5373. Then, Boc-Met-Val-Met-Gly-Ir(R1)_2_ is converted into compound **4** (NH_2_-Met-Val-Met-Gly-Ir(R1)_2_) using TFA in DCM in quantitative yield.

### Synthesis of compounds **5** and **6**

To a stirred solution of Boc-Trp(alloc)-Lys(alloc)-Tyr-COOH (250 mg, 0.32 mmol) and NH_2_-Met-Val-Met-Gly-Ir(R1)_2_ (445 mg, 0.353 mmol) in 20 mL of dry DCM were added triethylamine (Et_3_N) (93 μL, 0.64 mmol) and then EDCI (122 mg, 0.64 mmol). The reaction mixture was stirred overnight, and the reaction was followed by thin layer chromatography (TLC) (DCM/MeOH, 95/5). The reaction was stopped and washed with H_2_O (50 mL), 5% hydrochloric acid (HCl) (50 mL), and saturated sodium bicarbonate (NaHCO_3_) (50 mL). After drying over sodium sulphate (Na_2_SO_4_), the solvent was evaporated *in vacuo* to give a yellow solid which was purified using column chromatography to give 0.41 g of the pure Boc-Trp(alloc)-Lys(alloc)-Tyr-Met-Val-Met-Gly-Ir(R1)_2_ (**5**) as yellow crystals in good yield (63%).

To a stirred suspension of Boc-Trp(alloc)-Lys(alloc)-Tyr-Met-Val-Met-Gly-Ir(R1)_2_ (**5**) (30 mg, 0.014 mmol) and 10% Pd/C (10 mg) in 5 mL of MeOH was added triethylsilane (0.1 mL, 0.14 mmol) drop-wise under argon. After completion of reaction (TLC), the mixture was filtered through celite, and the filtrate was concentrated under vacuum. The crude product was purified by short silica gel column chromatography, eluting with 10% MeOH-DCM, to give 20 mg of 6 as light-yellow solid with a yield of 73%, and an overall yield of 43%. Iridium(iii) complex **6**: ^1^H NMR (400 MHz, DMSO) *δ* 10.85 (s, 1H), 9.79 (d, *J* = 5.7 Hz, 1H), 9.51 (d, *J* = 6.0 Hz, 1H), 8.26 (d, *J* = 7.5 Hz, 1H), 8.17 (d, *J* = 8.2 Hz, 1H), 8.08 (m, 1H), 8.01 (d, *J* = 7.6 Hz, 1H), 7.98 (s, 1H), 7.95 (s, 1H), 7.87 (s, 2H), 7.78 (d, *J* = 8.2 Hz, 1H), 7.72 (d, *J* = 7.9 Hz, 1H), 7.57 (d, *J* = 7.0 Hz, 2H), 7.44 (m, 1H), 7.30 (s, 1H), 7.21 (s, 2H), 7.09 (s, 1H), 7.02 (s, 2H), 6.95 (s, 2H), 6.88 (m, 1H), 6.82 (m, 1H), 6.75 (m, 1H), 6.68 (m, 1H), 6.23 (d, *J* = 7.1 Hz, 1H), 5.98 (dd, *J* = 17.2, 10.0 Hz, 1H), 5.84 (d, *J* = 17.1 Hz, 1H), 5.64 (d, *J* = 7.4 Hz, 1H), 5.36 (s, 1H), 4.47 (s, 1H), 4.42 (s, 1H), 4.20 (s, 1H), 4.12 (s, 2H), 3.57 (d, *J* = 6.3 Hz, 2H), 2.98 (s, 3H), 2.87 (s, 2H), 2.41 (d, *J* = 7.2 Hz, 2H), 2.00 (s, 5H), 1.82 (s, 2H), 1.28 (s, 7H), 1.21 (s, 3H), 1.12 (s, 2H), 0.83 (s, 5H). HRMS: calcd. for C_82_H_94_F_6_IrN_14_O_10_ S_2_P [M – PF_6_]^+^: 1691.634757 found: 1692.1516.

### Materials and cell lines

All chemicals were purchased from Sigma-Aldrich and were used as received. Lipofectamine™ 3000 reagent was purchased from Invitrogen (Carlsbad, CA, USA). Fetal bovine serum (FBS) and Dulbecco's Modified Eagle's Medium (DMEM) were purchased from Gibco BRL (Gaithersburg, MD, USA).

### Cytotoxicity assay

HUVEC cells were seeded in 96-well plates at a density of 5000 cells per well. After 12 h incubation, the cells were incubated with complex **6** at the indicated concentrations for 48 h. After incubation, 10 μL of 5 mg mL^–1^ MTT reagent was added to each well for 4 h. 100 μL of DMSO was then added to each well, and the intensity of absorbance was determined using a SpectraMax M5 microplate reader at a wavelength of 570 nm.

### Cell imaging

HUVEC cells were seeded into a glass-bottomed dish (35 mm dish with 20 mm wells) and incubated for 24 h. Complex **6** was added to cells for indicated time periods or concentrations, followed by washing with phosphate-buffered saline (PBS) three times. The luminescence imaging of complex **6** in cells was carried out using a Leica TCS SP8 confocal laser scanning microscope system. The excitation wavelength was 488 nm.

### FPR2 knockdown assay

HUVEC cells were seeded in 6-well plates at about 80% confluence in DMEM for 12 h. Lipofectamine™ 3000 reagent and siRNA were gently mixed in FBS-free DMEM medium. After 15 min incubation at room temperature, 500 μL of siRNA-lipid complex was directly added to cells in 1.5 mL DMEM culture medium. Then, HUVEC cells were incubated at 37 °C in a CO_2_ incubator for 48 h before use.

### Immunoblotting

HUVEC cells were washed twice with pre-chilled PBS after knockdown treatment, and harvested in lysis buffer. The protein concentration was determined by using the BCA assay. Total proteins were separated by SDS-polyacrylamide gel electrophoresis and then transferred onto polyvinylidene difluoride membranes (Millipore). After incubation with blocking buffer for 1 h at r.t., the membranes were incubated with primary antibodies at 4 °C overnight and secondary antibodies for 1 h incubation at room temperature. The protein bands were then stained with ECL Western Blotting Detection Reagent (GE Healthcare) and visualized using the ChemiDocTM MP Imaging System.

### *In vitro* wound migration assay

HUVEC cells were seeded in 6-well plates at about 90% confluence in DMEM overnight. The cells were scratched along the diameter of the well. The monolayers of HUVECs were scratch wounded with a 200 μL pipette tip, and then washed twice with serum-free medium and further incubated in DMEM medium with 1% serum. Images of the wounded monolayer of HUVECs were taken at 12 and 24 h after treatment with complex **6** by using an Olympus microscope system.

## Conclusions

Generally, luminescent probes are classified into three types: “always-on”, “turn-on” or “turn-off”, based on their luminescence behavior upon interaction with analytes.^56^,^57^ Always-on (permanent) or turn-on luminescent probes are preferred because of their lower target-to-background ratio, while “turn-off” probes may suffer from luminescence quenching by energy transfer mechanisms due to interactions with biological interferents.^56^,^58^ In this work, we designed and synthesized a WKYMVm-conjugated iridium(iii) complex (**6**) as a permanent FPR2 imaging probe. This probe showed a long luminescence lifetime of 4.62 μs and good photostability. In luminescence imaging experiments, the probe “lighted up” FPR2-expressing HUVEC cells in a dose- and time-dependent manner. WRW4 agonist competition and siRNA knockdown experiments provided further evidence that the probe selectively targeted FPR2 in HUVEC cells. Importantly, the probe was relatively nontoxic at the concentrations required for cell imaging. FPR2 silencing decreased the migration capacity of HUVEC cells in the scratch assay, verifying the role of the FPR2 in epithelial cells. Significantly, our probe decreased the migration of HUVEC cells both in the absence and presence of the FPR2 agonist LXA4, revealing its FPR2 inhibitory role in living HUVEC cells. These results show that the peptide-conjugated probe not only effectively recognizes FPRs, but also regulates FPR2-mediated functions in living cells. To the best of our knowledge, this is the first long-lived probe reported for FPR2 cellular imaging, and we anticipate that it could serve as a good starting point for the development of other kinds of luminescent FPR2 probes or possibly new drugs for the treatment of inflammatory diseases.

## Conflicts of interest

There are no conflicts to declare.

## Supplementary Material

Supplementary information
