# Supplementary material for: A long-lived peptide-conjugated iridium(iii) complex as a luminescent probe and inhibitor of the cell migration mediator, formyl peptide receptor 2
Source: Chem Sci. 2018 Oct 1;9(43):8171–7. doi: 10.1039/c8sc02733a (PMC6256120; doi:10.1039/c8sc02733a)
Supplement: Supplementary file 1 [file SC-009-C8SC02733A-s001.pdf]

Electronic Supplementary Material (ESI) for Chemical Science.
This journal is © The Royal Society of Chemistry 2018

Supporting Information

A long-lived peptide-conjugated iridium(III) complex as a luminescent probe and inhibitor of the cell migration mediator, formyl peptide receptor 2

Kasipandi Vellaisamy,‡[a] Guodong Li,‡[b] Wanhe Wang,‡[a] Chung-Hang Leung,*[b] Dik-Lung Ma*[a]

[a] *Department of Chemistry, Hong Kong Baptist University, Kowloon Tong, Hong Kong, China. E-mail: edmondma@hkbu.edu.hk*

[b] *State Key Laboratory of Quality Research in Chinese Medicine, Institute of Chinese Medical Sciences, University of Macau, Macao, China. E-mail: duncanleung@umac.mo*

Material

General Experimental

Mass spectrometry was performed at the Mass Spectroscopy Unit at the Department of Chemistry, Hong Kong Baptist University, Hong Kong (China). Deuterated solvents for NMR purposes were obtained from Armar and used as received. ^{1}H and ^{13}C NMR were recorded on a Bruker Avance 400 spectrometer operating at 400 MHz (^{1}H) and 100 MHz (^{13}C). ^{1}H and ^{13}C chemical shifts were referenced internally to solvent shift ($CDCl_3$−*d*: ^{1}H, 7.26, ^{13}C, 77.16; MeOD−d_4: ^{1}H, 3.31, ^{13}C, 1.32, 118.26; DMSO-d_6: ^{1}H, 2.50, ^{13}C, 39.52; acetone-d_6: ^{1}H, 2.05, ^{13}C, 29.8). Chemical shifts are quoted in ppm, the downfield direction being defined as positive. Uncertainties in chemical shifts are typically ±0.01 ppm for ^{1}H and ±0.05 for ^{13}C. Coupling constants are typically ±0.1 Hz for ^{1}H-^{1}H and ±0.5 Hz for ^{1}H-^{13}C couplings. The following abbreviations are used for convenience in reporting the multiplicity of NMR resonances: s, singlet; d, doublet; t, triplet; q, quartet; m, multiplet; br, broad. All NMR data were acquired and processed using standard Bruker software (Topspin).

Purity experiment. The purity of complex **6** was examined by Agilent 1200 high-performance liquid chromatography (HPLC) system using an Agilent C18 column (4.6 mm × 250 mm, 5 μm). The sample volume injected was 20 μL with a flow rate of 1.0 mL/min. Mobile phase A is Milli-Q H_2O (with 0.1% *v*/*v* trifluoroacetic acid (TFA)) and mobile phase B is acetonitrile (with 0.1% *v*/*v* TFA), respectively. The mobile phase gradient was initially set for 10% acetonitrile, 10–45% acetonitrile from 0 min to 10 min, 45–75% acetonitrile from 10 min to 15 min, and ended up as a 95% acetonitrile over the 25 min run time. UV absorbance was monitored at 254 nm.

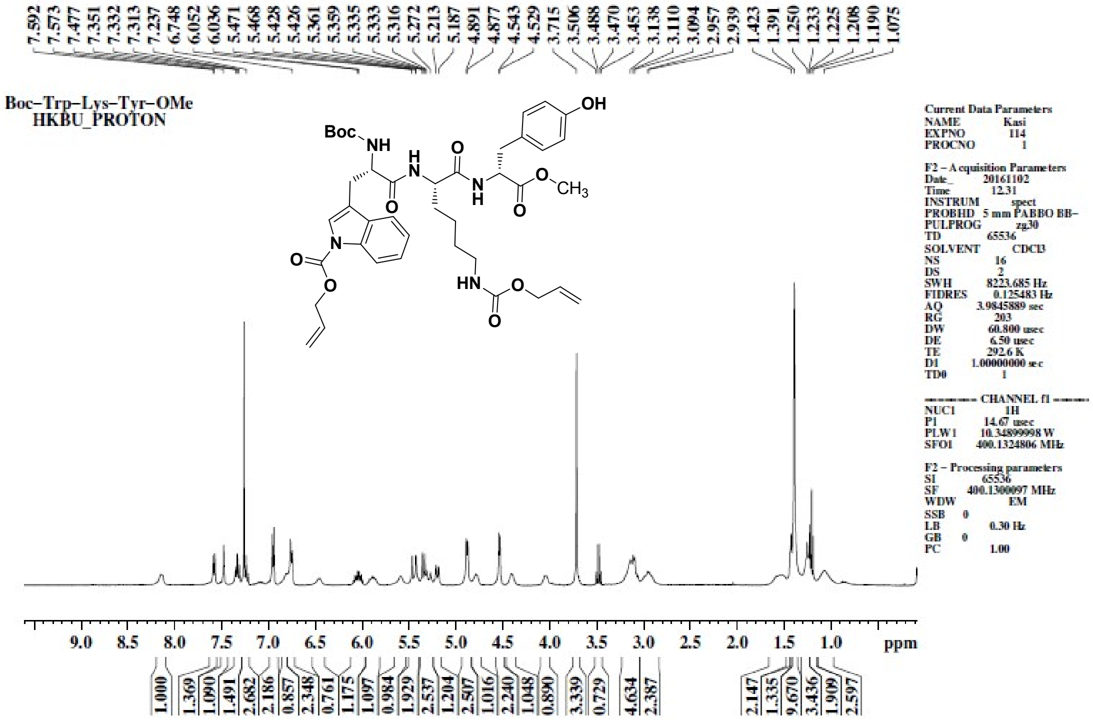

Figure S1. ^{1}H NMR spectrum of compound **1**.

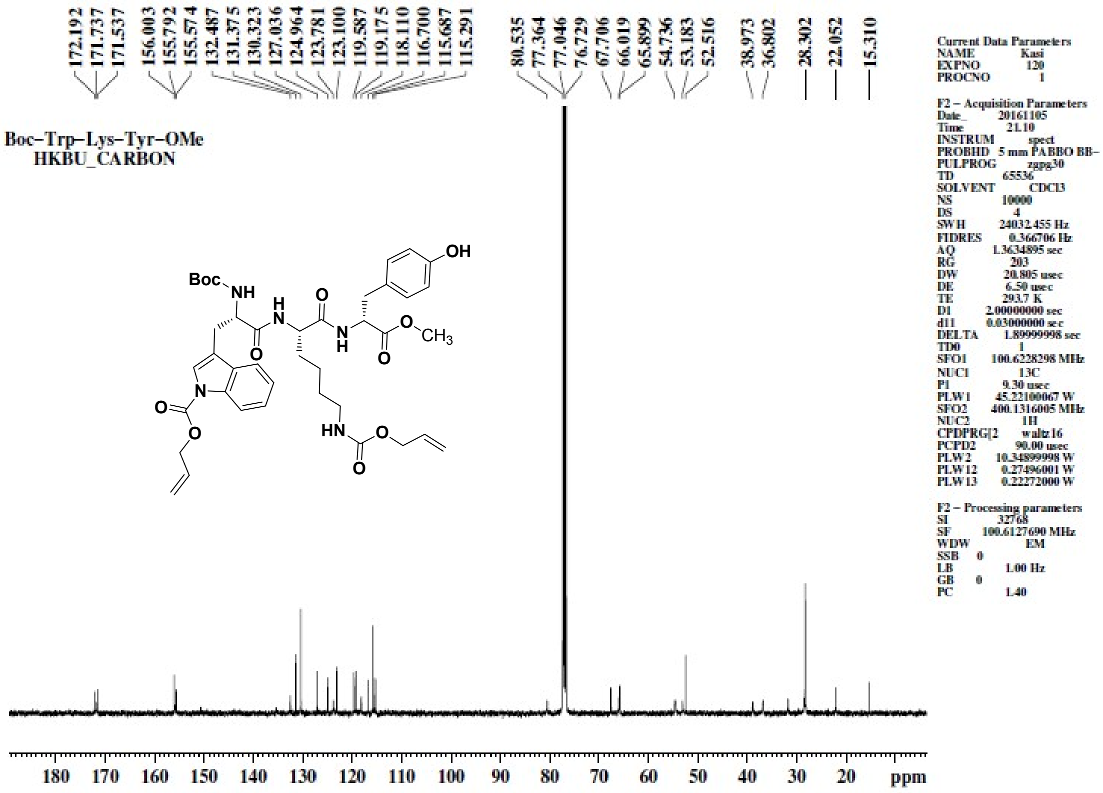

Figure S2. ^{13}C NMR spectrum of compound **1**.

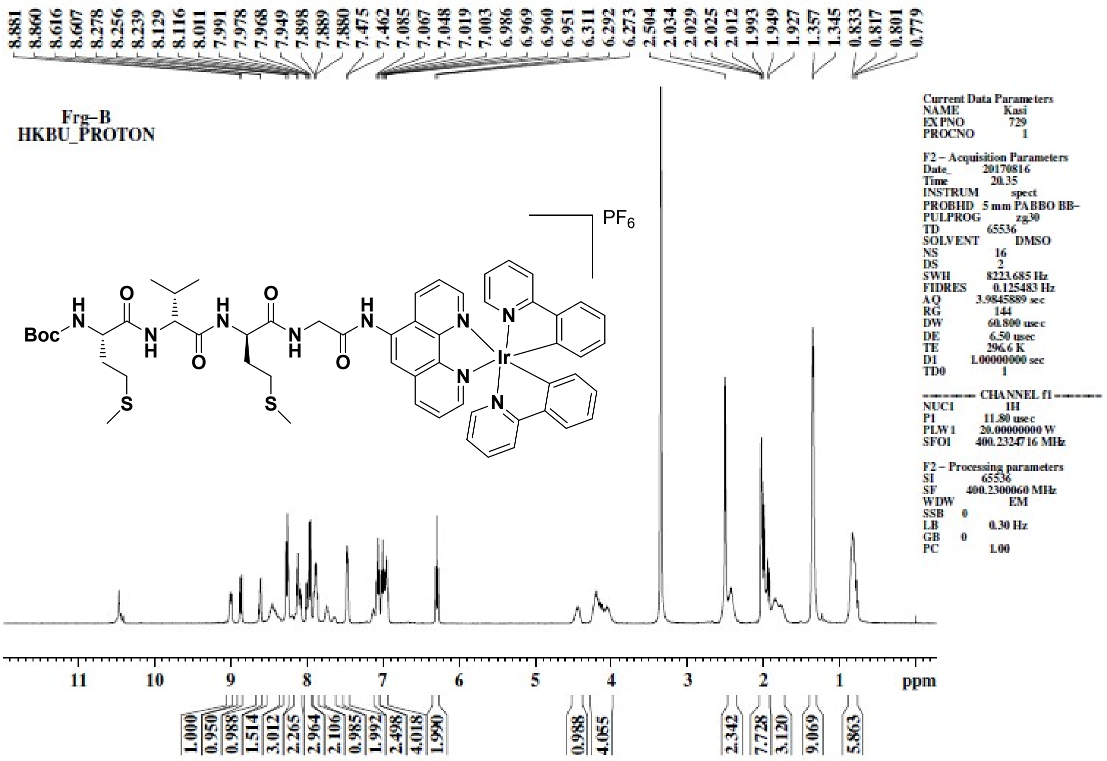

Figure S3. ^{1}H NMR spectrum of compound **2**.

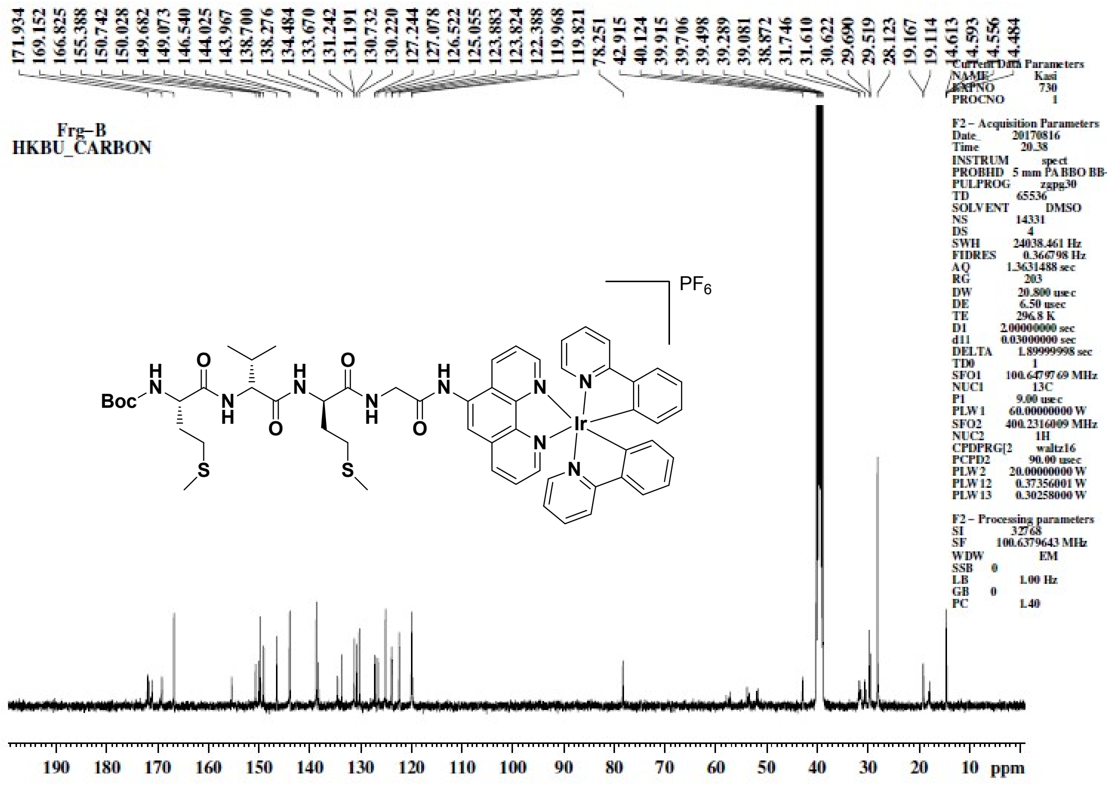

Figure S4. ^{13}C NMR spectrum of compound **2**.

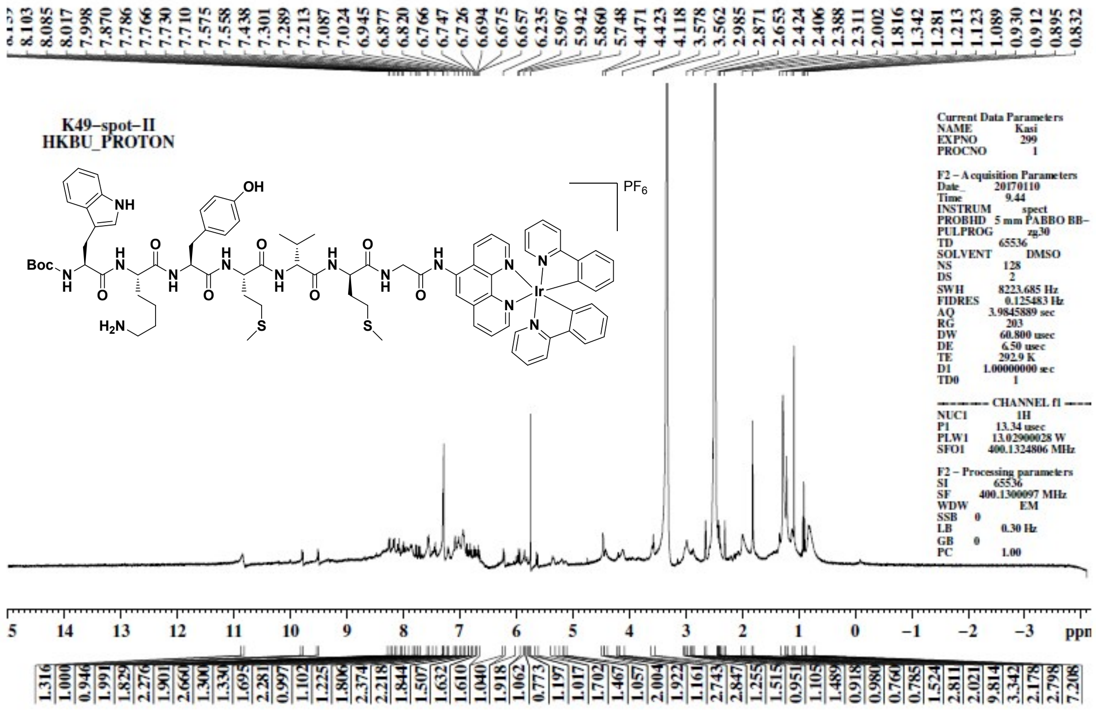

Figure S5. ^{1}H NMR spectrum of iridium(III) complex **6**.

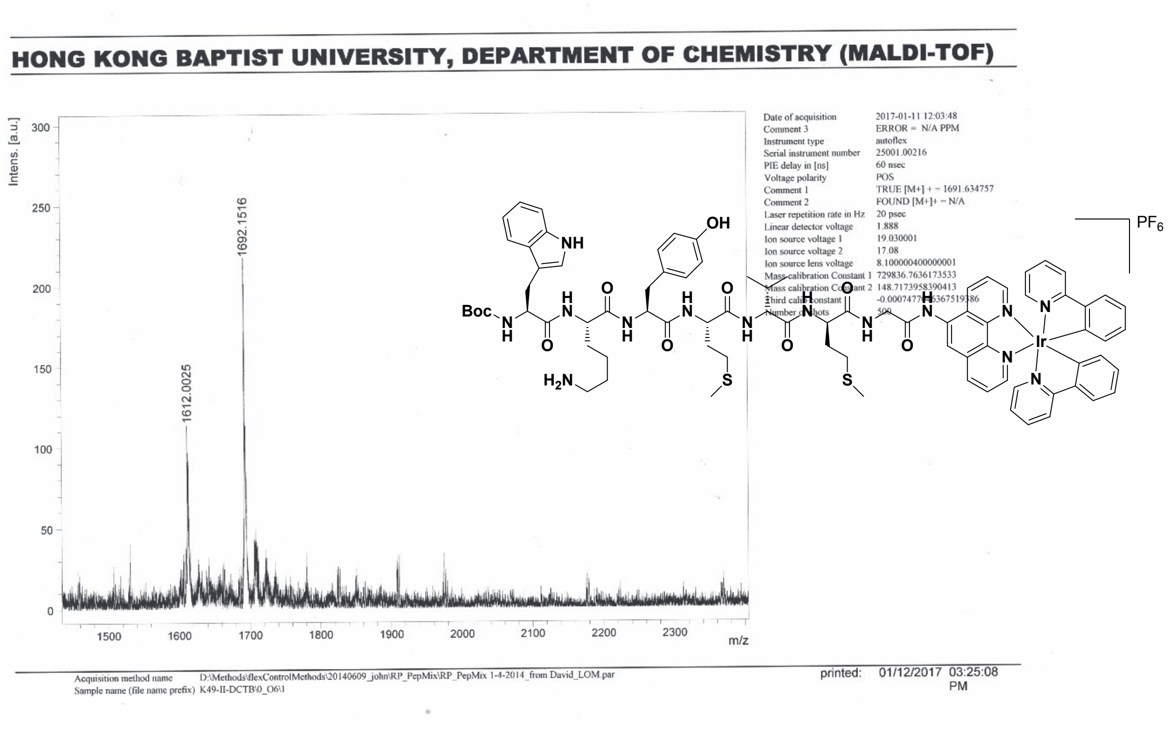

Figure S6. MALDI-TOF spectrum of iridium(III) complex **6**.

HPLC purity of iridium(III) complex 6

```
Data File D:\DATA\WANHE\IR_EGFR01.D
Sample Name: Ir_EGFR

    =====================================================================
    Acq. Operator   : JLJ                            Seq. Line :   1
    Acq. Instrument : Instrument 1                    Location : Vial 2
    Injection Date  : 1/15/2017 9:04:42 PM                 Inj :   1
                                                    Inj Volume : 20 µl
    Acq. Method     : D:\WANHE\91-64.M
    Last changed    : 1/15/2017 8:57:51 PM by JLJ
    Analysis Method : D:\TS-1-20170118-2.M
    Last changed    : 1/18/2017 2:07:02 PM by zy
```

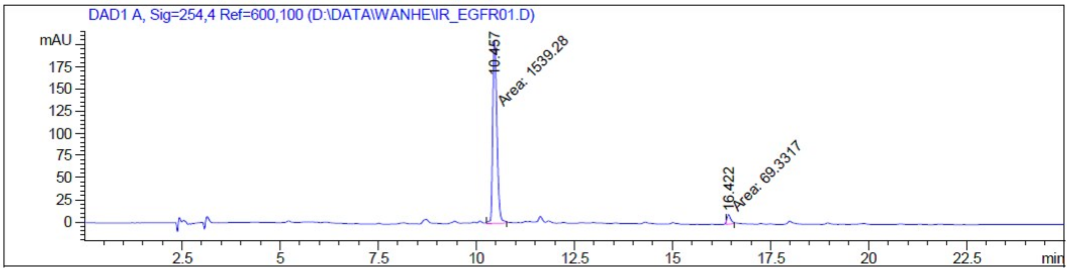


```
Sorted By             :      Signal
Multiplier:                  :      1.0000
Dilution:                    :      1.0000
Use Multiplier & Dilution Factor with ISTDs


Signal 1: DAD1 A, Sig=254,4 Ref=600,100

Peak RetTime Type  Width     Area      Height     Area
  #    [min]       [min]   [mAU*s]     [mAU]        %
----|-------|----|-------|----------|----------|--------|
   1  10.457 MM    0.1244 1539.28137  206.17451  95.6900
   2  16.422 MM    0.1060   69.33173   10.89934   4.3100

Totals :                  1608.61310  217.07386
```

Figure S7. HPLC spectrum of iridium(III) complex **6**.

Table S1. Photophysical properties of iridium(III) complex **6**.

Complex	Quantum yield	λ_{emi} / nm	Lifetime / µs	UV/Vis absorption λ_{abs} / nm (ε / dm^3 mol^{-1})
**6**	0.241	576	4.62	255 (0.45×10^5)

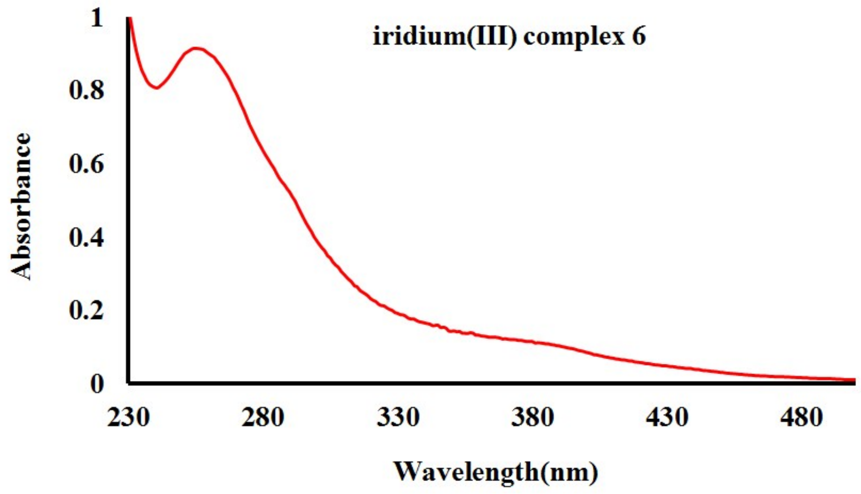

Figure S8. UV/Vis absorption of iridium(III) complex **6** (20 μM) in acetonitrile at 298 K.

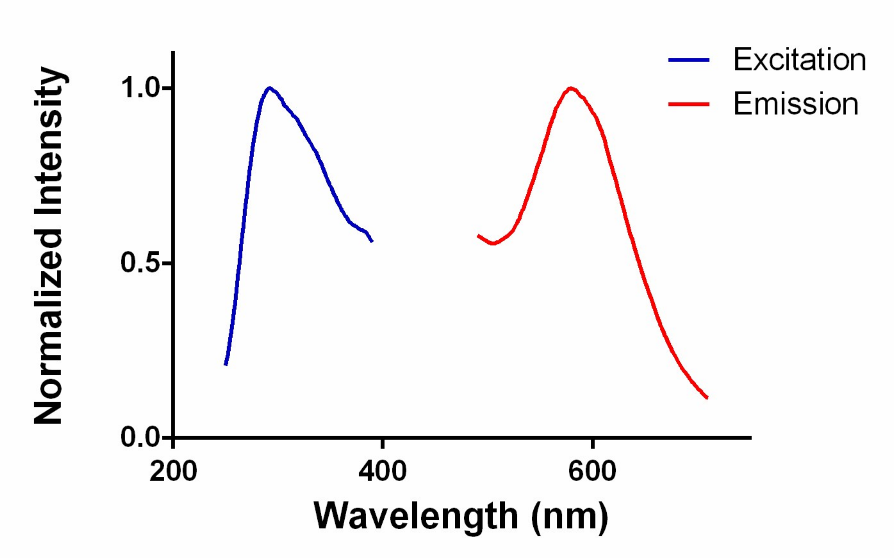

Figure S9. Maximum excitation and emission spectra of complex **6** (10 μM) in DMSO.

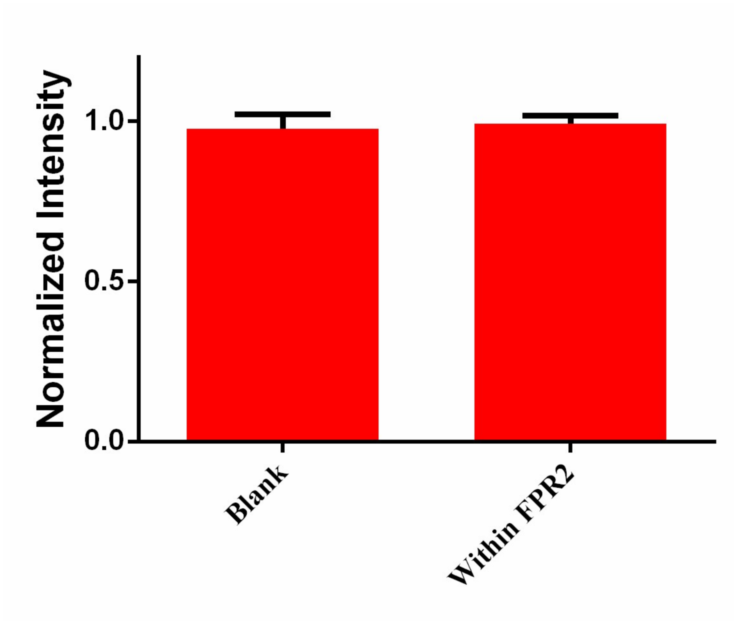

Figure S10. Luminescence of complex **6** (10 μM) in the absence and presence of 0.3 μg/mL FPR2.

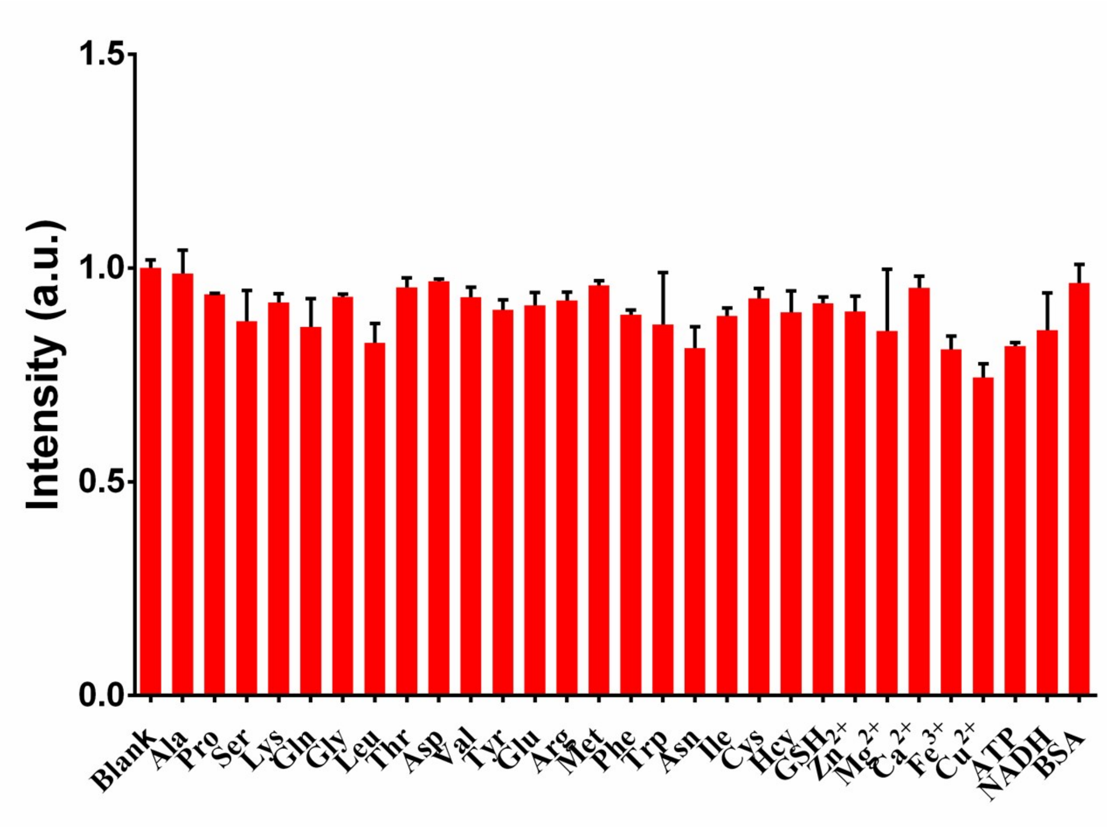

Figure S11. Luminescent response of complex **6** (10 μM) to various analytes in PBS (7.4).

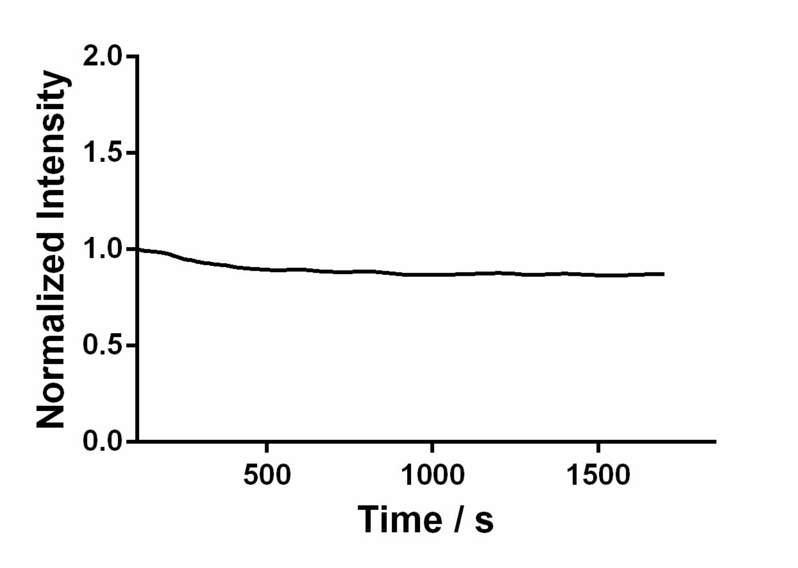

Figure S12. Time course of luminescence of complex **6** (10 μM) in PBS (pH 7.4) over 1800 s under continuous irradiation at 365 nm.

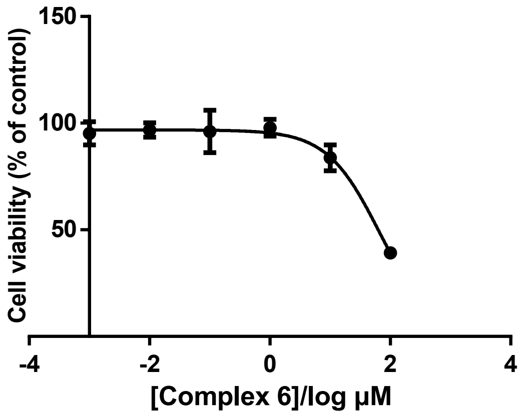

Figure S13. HUVEC cells were treated with different concentrations of complex **6** for 48 h. Complex **6** exhibited an IC_{50} value of 63.09 μM.

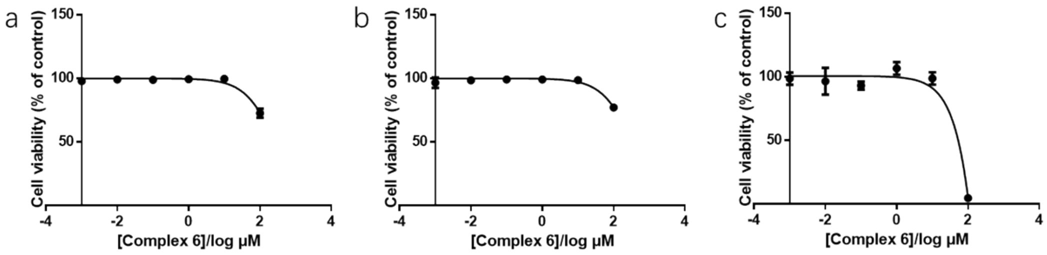

Figure S14. (a) LO2, (b) HeLa, and (c) MDA-MB-231 were treated with different concentrations of complex **6** for 48 h, and cell viability was determined using the MTT assay. Complex **6** exhibits IC_{50} value of >100, >100, and 52.48 μM, respectively. Error bars represent the standard deviations of the results from three independent

experiments.

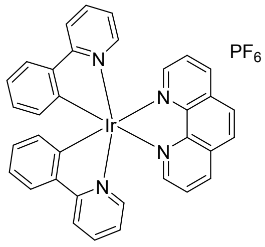

S1

Figure S15. Chemical structure of complex **S1**.

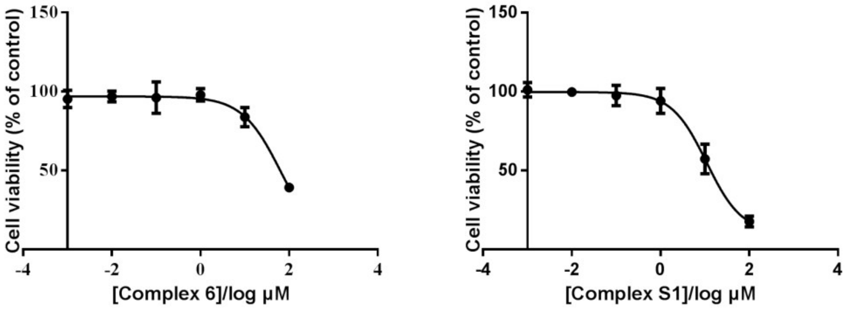

Figure S16. HUVEC cells were treated with different concentrations of complexes **6** or **S1** for 48 h, and cell viability was determined using the MTT assay. Complex **6** exhibited an IC_{50} value of 63.09 μM, while complex **S1** exhibited an IC_{50} value of 14.13 μM. Error bars represent the standard deviations of the results from three independent experiments.

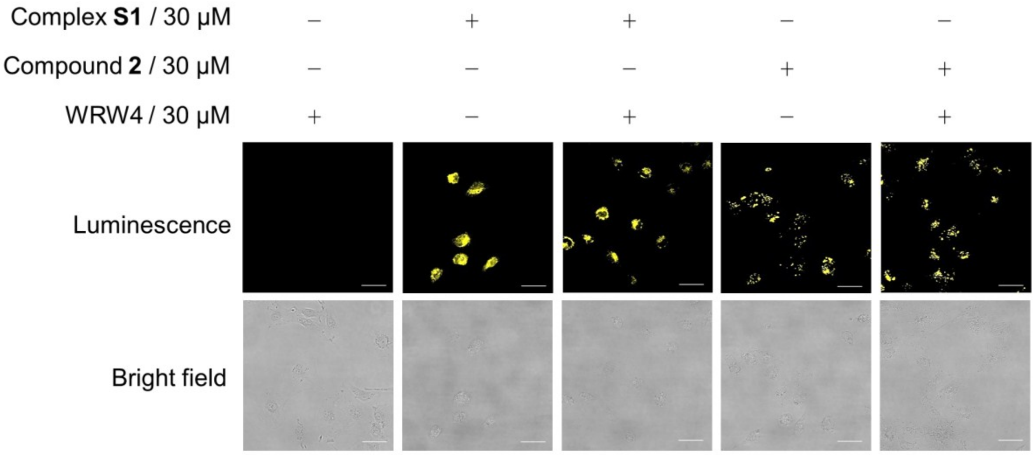

Figure S17. HUVEC cells were treated with WRW4/vehicle at different concentrations for 1 h, followed by staining with complexes **S1** or **2** (30 μM) for 1 h. Scale bar = 100 μm.

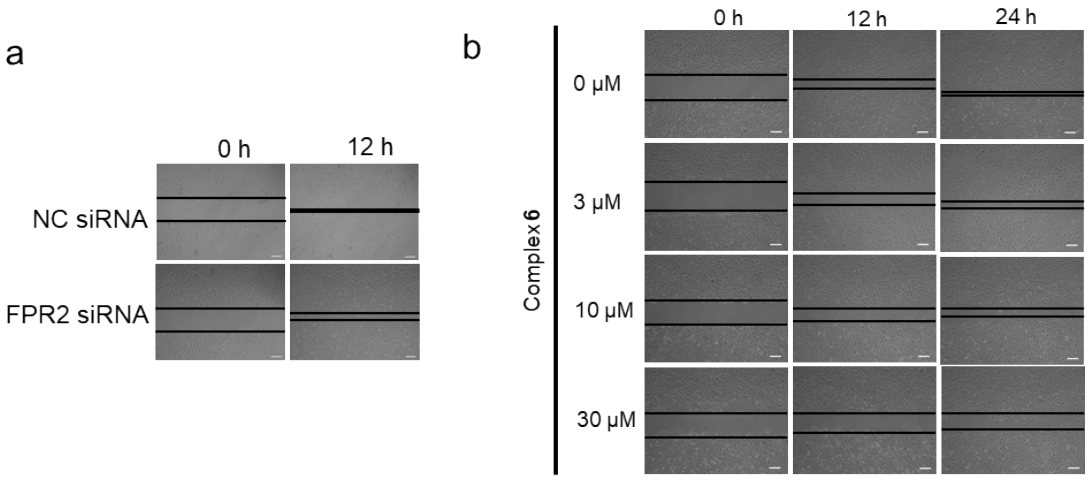

Figure S18. (a) Images of the wounded monolayer of HUVECs taken at 12 h after treatment with negative control (NC) siRNA or FPR2 siRNA. The horizontal lines indicate the wound edge. Scale bar = 200 μm. (b) Images of the wounded monolayer of HUVECs were captured at 0, 12 and 24 h after complex **6** treatment (0, 3, 10 and 30 μM). The horizontal lines indicate the wound edge. Scale bar = 200 μm.
